# Supplementary material for: Myomatrix arrays for high-definition muscle recording
Source: bioRxiv. 2023 Sep 19:2023.02.21.529200. Originally published 2023 Feb 22. Preprint. [Version 2] doi: 10.1101/2023.02.21.529200 (PMC9980060; doi:10.1101/2023.02.21.529200)
Supplement: 1 [file NIHPP2023.02.21.529200V2-supplement-1.pdf]

bioRxiv preprint doi: https://doi.org/10.1101/2023.02.21.529200; this version posted September 19, 2023. The copyright holder for this preprint (which was not certified by peer review) is the author/funder, who has granted bioRxiv a license to display the preprint in perpetuity. It is made available under aCC-BY 4.0 International license.

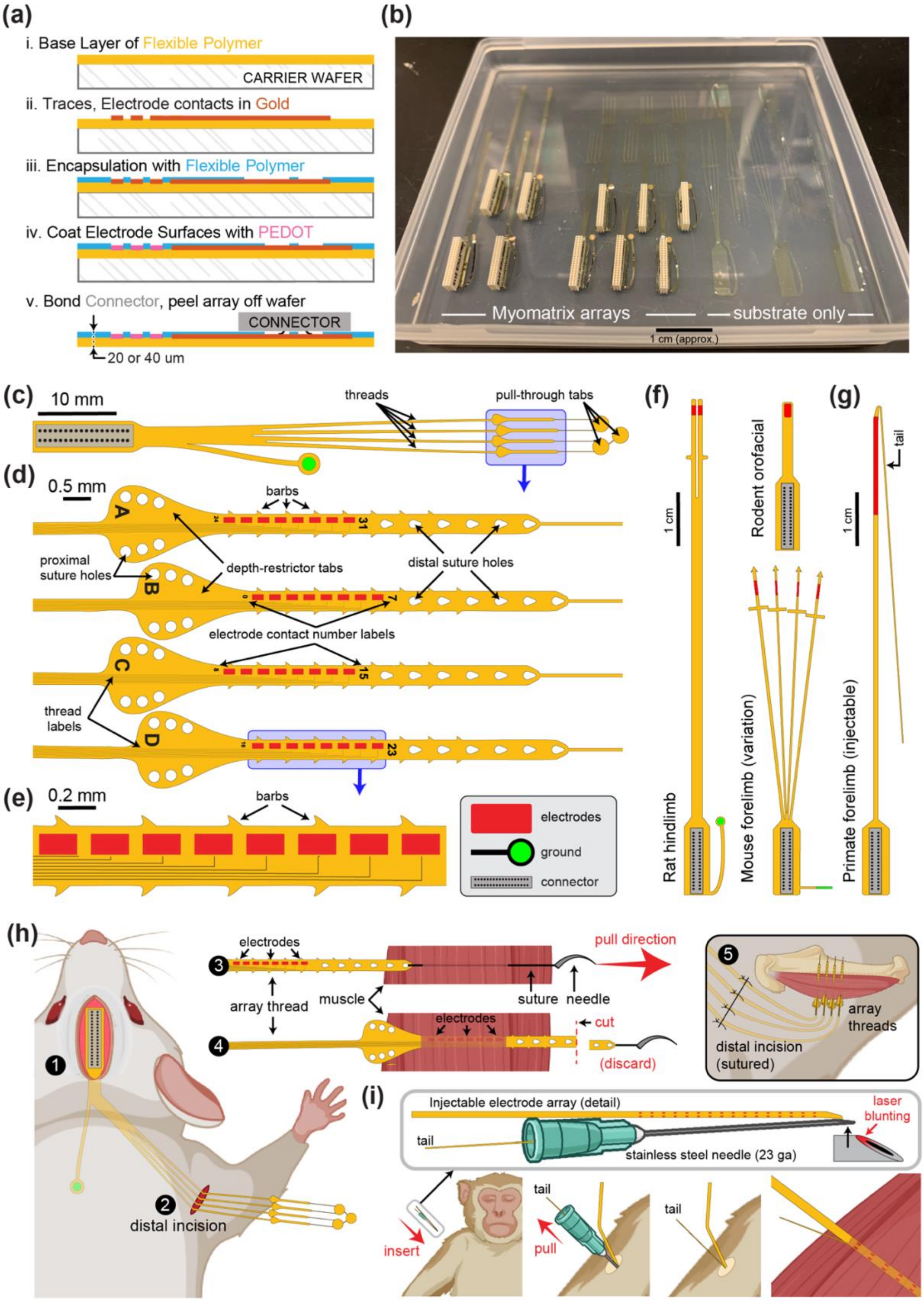

Supplemental Figure 1: Myomatrix fabrication, design variations, and implantation.

(a) Workflow for electrode array fabrication. Layers of insulating polymer (polyimide) and conductive metal (gold) are successively deposited on a carrier wafer to form a flexible, 20 or 40 μm thick electrode array of gold electrode contacts, which receive a surface treatment of PEDOT to improve recording properties (see Methods). Electrodes are connected via thin gold traces to a receiving pad for a high-density connector (Omnetics Inc.) which is then bonded to the array. The completed array is then peeled off the

bioRxiv preprint doi: https://doi.org/10.1101/2023.02.21.529200; this version posted September 19, 2023. The copyright holder for this preprint (which was not certified by peer review) is the author/funder, who has granted bioRxiv a license to display the preprint in perpetuity. It is made available under aCC-BY 4.0 International license.

carrier wafer. **(b)** Photo showing two different Myomatrix designs (left) as well as "blank" arrays comprised only of the flexible polyimide substrate for surgical practice and design optimization. **(c-e)** Expanded views of the electrode array also shown in **Figure 1** of the main manuscript text, which has four "threads" each bearing eight electrode contacts. This array design can be used for either acute or chronic recordings. For chronic implantation, the surgeon grasps the "pull-through tabs" when tunneling the threads subcutaneously. For intramuscular implantation in either acute or chronic settings, a needle is used to pull each thread through the target muscle. In this use case, the "depth-restrictor tabs" prevent the thread from being pulled any further into the muscle, thereby determining the depth of the electrode contacts within the muscle. **(d,e)** Detail views highlighting sub-millimeter features used to increase electrode stability within the muscle (barbs, suture holes) and labels to indicate which channel/thread labels have been implanted in which muscles. **(f,g)** Design variations. The fabrication process shown in **(a)** can easily be modified to alter size and shape of the electrode array. Each Myomatrix design in **f** and **g** has 32 electrode contacts. **(f)** Two array designs customized for chronic recording applications in different muscle groups in rodents. **(g)** Injectable array for recording forelimb muscles in nonhuman primates. **(h)** For chronic implantation in mice, the connector end of the array is attached to the skull using dental acrylic (1) and the flexible array threads are then routed subcutaneously to a small distal incision located near the targeted muscle or muscles (2). For intramuscular implantation, the surgeon secures each thread to a suture and needle, which are then inserted through the target area of muscle tissue (3). The surgeon then pulls the suture further through the muscle, eventually drawing the array thread into the muscles such that the depth-restrictor tabs prevent further insertion and ensure that the electrode contacts are positioned at the correct depth within the muscle (4). In contrast, for epimysial (as opposed to intramuscular) implantation, the array threads are sutured to the surface of the muscle fascia rather than being inserted with a suture needle. After all threads are secured to the muscle, the distal incision site is sutured closed (5). **(i)** For percutaneous insertion of injectable arrays, the array's thin "tail" is loaded into a modified hypodermic syringe [14,34]. During insertion, the tail is secured by bending it back over the plastic needle holder and securing it with either the surgeon's fingers or an additional syringe inserted into the cannula. After electrode array insertion the needle is gently pulled out of the muscle, leaving the electrode-bearing part of the array thread within the target muscle for the duration of the recording session. After recording, the electrode and tail are gently pulled out of the muscle together as with injectable fine-wire EMG [14].

bioRxiv preprint doi: https://doi.org/10.1101/2023.02.21.529200; this version posted September 19, 2023. The copyright holder for this preprint (which was not certified by peer review) is the author/funder, who has granted bioRxiv a license to display the preprint in perpetuity. It is made available under aCC-BY 4.0 International license.

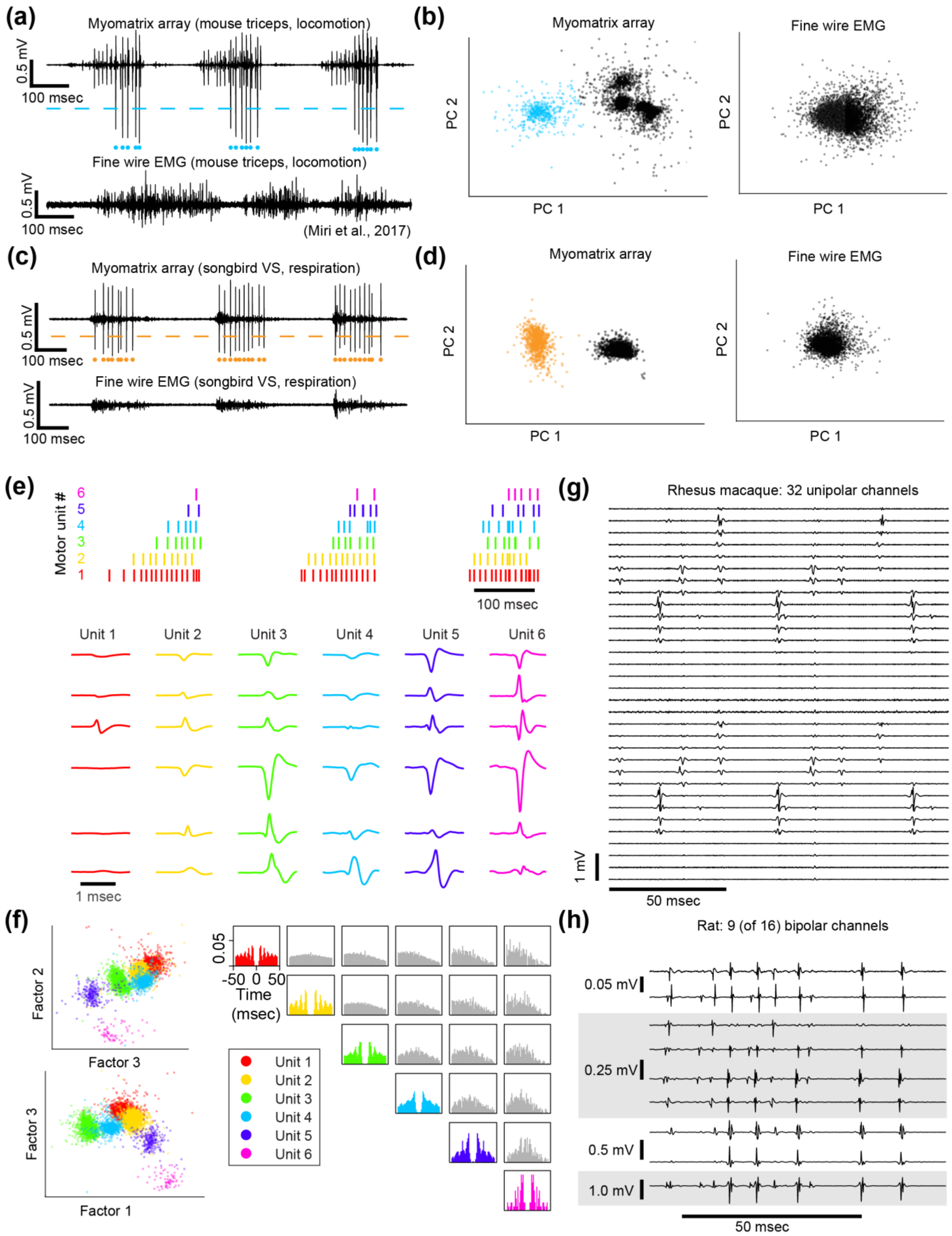

Supplemental Figure 2: Spike sorting

Action potential ("spike") waveforms from individual motor units can be identified ("sorted") using analysis methods that are commonly used to sort spikes from neural data. **(a-d)** Single channel spike sorting. In some cases, a single motor unit's spike will

bioRxiv preprint doi: https://doi.org/10.1101/2023.02.21.529200; this version posted September 19, 2023. The copyright holder for this preprint (which was not certified by peer review) is the author/funder, who has granted bioRxiv a license to display the preprint in perpetuity. It is made available under aCC-BY 4.0 International license.

dominate the recording on an individual Myomatrix channel, as shown in an example bipolar recording from mouse triceps during locomotion (**a**, top). In such cases, a simple voltage threshold (dashed line) can be used to isolate spike times of the largest recorded unit (blue dots) from a single channel. In contrast (**a,** bottom), fine-wire EMG typically does not yield isolated single units during active behaviors. **(b)** Single-channel spike sorting using principal components analysis (PCA) of the data shown in (a). Each data point in (b) represents a single voltage waveform represented in the dimensions defined by the first two principal components (PC1 and PC2) of the set of all spike waveforms. As described previously [22], k-means clustering can discriminate the waveforms from individual motor units (cyan dots in **a** and **b**) and waveforms from other motor units and/or background noise (black dots in **b**). If one of the clusters has less than 1% overlap with any other cluster (based on fitting each cluster with a 2D Gaussian as described previously) and displays an absolute refractory period (less than 1% of inter-spike intervals less than 1 msec), it is classified as a single unit [22]. When applied to the Myomatrix data in (a), PCA-based sorting method produced identical spike times as the thresholding method (cyan dots in **a**). In contrast, the same analysis applied to the fine-wire data shown in **a** did not produce any well-isolated clusters in PCA space (**b**, right), indicating that this method could not extract any single motor units. Myomatrix and fine-wire data shown in (a,b) are from the same datasets as the examples shown in main text Figure 1a,b. **(c,d)** Single-channel spike sorting applied to bipolar Myomatrix recordings from the ventral syringeal (VS) muscle, a songbird vocal muscle [20]. Here again, PCA-based sorting of Myomatrix data method produced identical spike times as the thresholding method (orange dots in **c** and **d**). In contrast, the same analysis applied to fine-wire data recorded from VS shown in **c** did not produce any well-isolated clusters in PCA space (**d**, right), Other plotting conventions for (c,d) are the same as for the mouse data in (a,b).

(e-h) Multichannel spike sorting using Kilosort. We used Kilosort version 2.5 [2,47] and custom MATLAB and Python code to sort waveforms into clusters arising from individual motor units. **(e)** Spike times (top) and mean waveforms (bottom) of six motor units recorded simultaneously from mouse triceps during locomotion (same dataset as **Fig. 1** in the main text). Mean waveforms for the six motor units (columns at bottom) are shown from six different EMG channels (rows) and illustrate the distinct pattern of spike waveforms across channel associated with the discharge of each identified motor unit. **(f)** Left, feature space projection of individual waveforms (colored dots), projected onto the space of singular values ("factors") that describe the space of all recorded waveforms. The clustering of waveforms from kilosort-identified units (colors) further illustrates the distinctness of voltage waveforms assigned to each of the identified motor units. Right, autocorrelograms (colors) and cross correlograms (gray) of the six motor units shown in (e). In addition to examining the consistency of each candidate motor unit's spike waveforms we also inspected autocorrelations to ensure that each identified unit showed an absolute refractory period (zero or near-zero autocorrelations at lag zero) and that cross-correlograms did not have strong peaks at zero lag (which might indicate the same motor unit being detected by multiple Kilosort clusters). **(g,h)** Myomatrix recordings from nonhuman primate and rat (unipolar and bipolar recordings respectively, same datasets as in main text **Fig. 3 and Fig. 2c**), respectively. These examples (along with the mouse data in main text **Fig. 1c**) highlight the finding that Myomatrix arrays typically record the same motor unit on multiple channels simultaneously. This redundancy is critical for Kilosort and related methods to isolate single motor unit waveforms, particularly when waveforms from multiple units overlap in time.

bioRxiv preprint doi: https://doi.org/10.1101/2023.02.21.529200; this version posted September 19, 2023. The copyright holder for this preprint (which was not certified by peer review) is the author/funder, who has granted bioRxiv a license to display the preprint in perpetuity. It is made available under aCC-BY 4.0 International license.

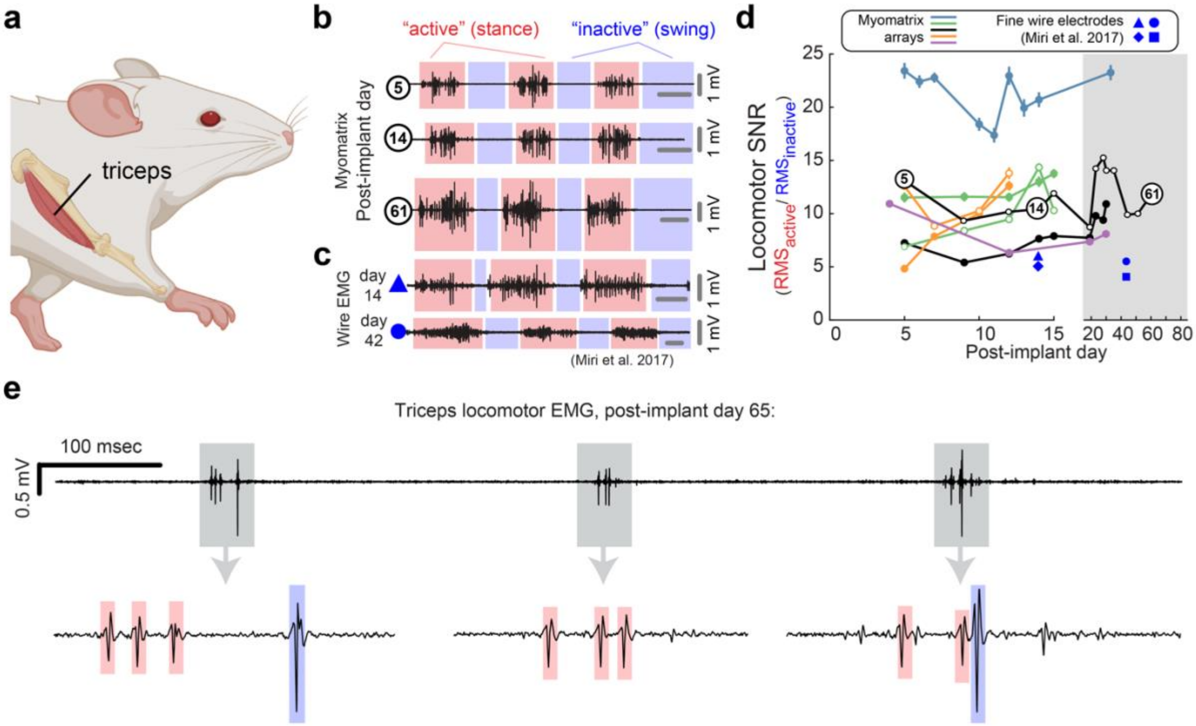

Supplemental Fig 3: Longevity of Myomatrix recordings

In addition to isolating individual motor units, Myomatrix arrays also provide stable multi-unit recordings of comparable or superior quality to conventional fine wire EMG. **(a,b)** Bipolar Myomatrix recordings from the triceps muscle of a mouse recorded during treadmill locomotion over a period 61 days. Colored regions in (b) highlight the "stance" phase (when the paw from the recorded forelimb is in contact with the treadmill surface) and "swing" phase (when the paw is lifted off the treadmill surface). To quantify changes in recording quality over time, we computed a "signal-to-noise ratio (SNR)" for each of each stride cycle as described previously [21]. Here, the "locomotor SNR" for each swing-stance-cycle is defined as the root mean square (RMS) amplitude of the multi-unit EMG signal during each single stance cycle divided by the RMS of the EMG signal during the immediately subsequent swing phase. **(c)** Fine-wire EMG data recorded from the triceps muscle during locomotion (reproduced with permission from [1]. Note that all horizontal gray bars in (b,c) represent 100 msec. **(d)** Mean +/- standard error of locomotor SNR across five mouse subjects implanted with Myomatrix arrays. Filled symbols indicate EMG implantation in the right triceps muscle, unfilled symbols indicate EMG implantation in the left triceps. The black trace with unfilled symbols represents the animal whose data are also shown in panel (b). In some cases, error bars are hidden behind plotting symbols. Blue symbols indicate the locomotor SNR from the fine-wire data from [1], with each symbol representing a single day's recording from one of four individual mice. SNR values from Myomatrix arrays are significantly greater than those from fine-wire EMG, both when all data shown in (d) are pooled and when only data from day 14 are included (2-sample KS-test, p=0.002 and p=0.038, respectively). (e) Although individual motor units were most frequently recorded in the first two weeks of chronic recordings (see main text), Myomatrix arrays also isolate individual motor units after much longer periods of chronic implantation, as shown here where spikes from two individual motor units (colored boxes in bottom trace) were isolated during locomotion 65 days after implantation. This bipolar recording was collected from the subject plotted with unfilled black symbols in panel (d).
